# Achiral SFC separations: Gold standard for the next generation of nontarget screening

**DOI:** 10.1002/ansa.202000109

**Published:** 2020-12-02

**Authors:** Stefan Bieber, Thomas Letzel

**Affiliations:** ^1^ AFIN‐TS GmbH (Analytisches Forschungsinstitut für Non‐Target Screening GmbH) Am Mittleren Moos 48 Augsburg 86167 Germany

In a recent publication, Pilařová et al. discussed the question if supercritical fluid chromatography (SFC) coupled to mass spectrometry (MS) might be an option for the analysis of complex samples and concluded that SFC has great potential.^1^ We fully support this statement and even would like to extend it to “SFC is an important separation technique and offers many benefits for the mass spectrometric analysis of very complex samples.” These benefits enable SFC to be a perfect technique for novel analytical strategies like the so‐called “mass spectrometric nontarget screening” (NTS).

## 1 UNFOCUSSED ANALYSIS OF COMPLEX SAMPLES

NTS is an emerging screening strategy, which can widely be used to address various analytical challenges. For instance, it can be applied to analyze samples on a molecular level and to gain information about contained (unexpected or unknown) compounds, to compare samples, to assess differences and commonalities, and to detect molecular trends among different samples.^2^

This strategy relies on powerful analytical techniques, mainly liquid chromatography (LC) coupled to accurate high‐resolution MS and well‐designed robust data evaluation concepts. To gain a broad perspective on samples in NTS, it is important to achieve a most comprehensive analysis of several compound classes. This highlights the necessity of using powerful and reliable separation techniques. In our perspective, SFC coupled to MS(/MS) can play an important role in NTS and become the next gold standard as chromatographic separation technique in this field. So far, reversed phase LC (RPLC) is the mostly used separation technique in this field, especially in analyzing (environmental) water samples. RPLC is a well‐established and robust separation technique, but the polarity of separable compounds is restricted to polar and nonpolar compounds. It is not suitable for the separation of (very) polar compounds.^3^ As a consequence in 2016, an "analytical gap" was postulated for the analysis of very polar compounds.^4^ Although there are several analytical techniques, suitable for the analysis of polar to very polar compounds, such as hydrophilic interaction chromatography (HILIC) or capillary chromatography (CE), these techniques hardly can separate nonpolar compounds. In 2017, the application of a serial coupling of RPLC and HILIC for the analysis of (environmental) water samples was presented.^3^ Furthermore, until now we used this system in our labs for more than 7 years in routine and have thoroughly assessed its strengths and weaknesses.^5^ So, the so‐called "analytical (polarity) gap" was closed very fast using this analytical set‐up and extended the analytical range of separable compounds so far. On the one hand, this allows the reproducible and robust separation and simultaneous analysis of nonpolar, polar, and very polar compounds in one experimental run. On the other hand, separations with this system take up to 35 minutes so far and due to the requirement of consequent HILIC re‐equilibration the total run‐time of the serial coupling adds up to nearly 1 hour. Further, peak widths in HILIC can be broader in this set‐up than those obtained from RPLC and this sometimes reduces the column plates and the sensitivity in MS detection. Most important, the consumption of potentially harmful organic solvents in the mobile phase, mostly acetonitrile, is rather high. Nevertheless, this system provides benefits by significantly extending the polarity of separations, the option to assess compound polarity, as well as great robustness and reproducibility.^4^

**FIGURE 1:**
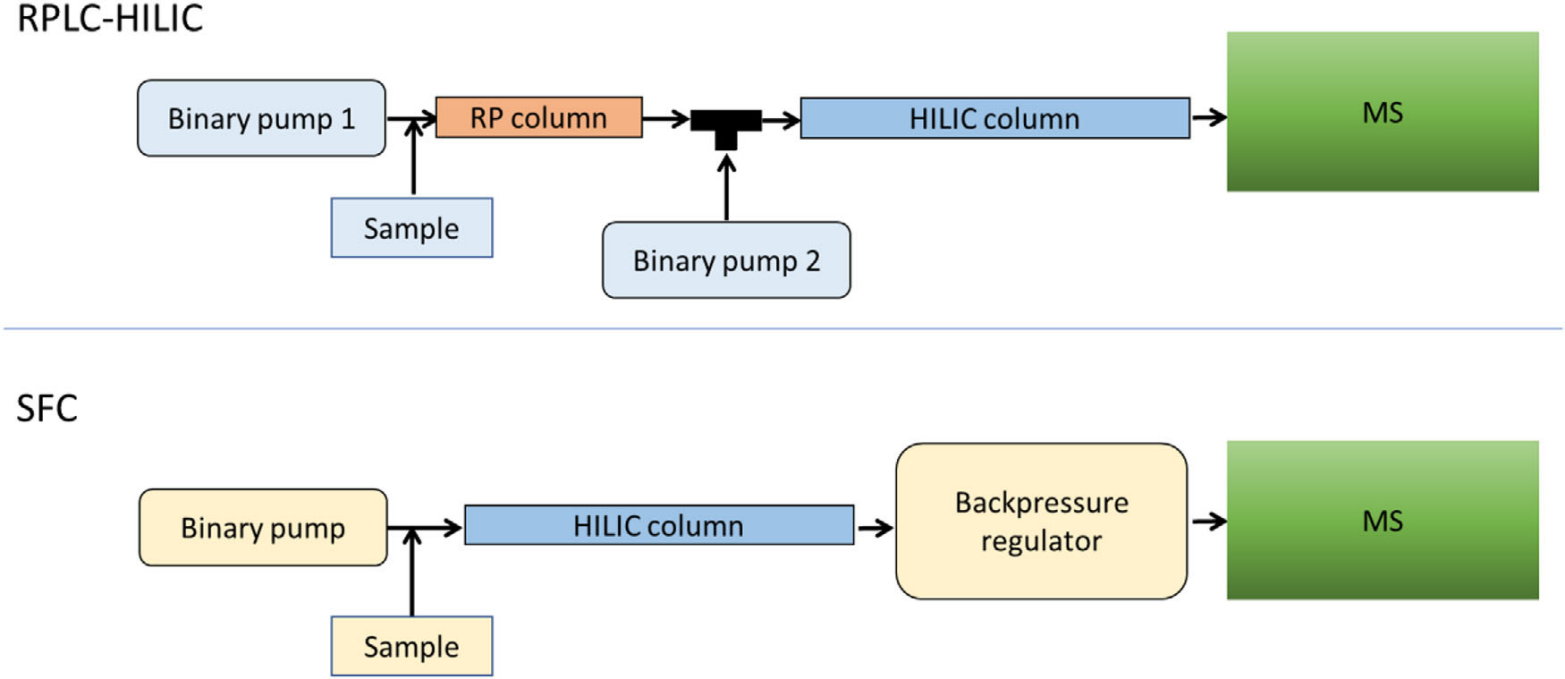
Instrumental set‐ups for a serial coupling of RPLC and HILIC and SFC, both coupled to MS detection. For further details, please see the correspondingly reference.^3^

When we started with SFC, we tested it as an orthogonal separation technique to LC. Therefore, we also used normal phase separation mode phases and compared them directly with the serial coupling of RPLC and HILIC (Figure 1).

## 2 CHALLENGES AND OPPORTUNITIES IN SFC METHOD DEVELOPMENT

However, setting up a suitable separation method was not easy at first. SFC separations are influenced by several factors, which are not present or adjustable in LC, such as the backpressure, which can be set at the backpressure regulator, or the compressibility of the mobile phase, which impacts mobile phase density. These factors can impact retention and selectivity and so, changes of a factor might have significant effects for the behavior of compounds with the stationary phase.^6^ In addition, there is currently limited knowledge about retention mechanisms. At the moment, when establishing a new separation method, it is necessary to screen several different stationary phases and co‐solvents in order to find a suitable combination for robust separations. Therefore, well‐applicable classification schemes for stationary phase have been presented,^7^ but method development in SFC remains more laborious than in LC. However, SFC provides the advantage to combine different stationary phases in an effective and easy way. We could show that the coupling of different columns can be a helpful tool in systematic method development and further increase the range of separable compounds.^8^ This concept can be applied if a set of compounds cannot be separated sufficiently by a specific column. Thus, it is possible to couple another stationary phase with a different selectivity and to achieve a separation with combined characteristics of both stationary phases. The selectivity of the combined stationary phases will not be a combination, but a sequential add‐up. In SFC, the ‘additive’ effect is possible using the POPLC style with isocratic mobile phases like in classical LC,^9^ as well as with mobile phase gradients.^8^

In RPLC and HILIC there is a good correlation of retention time and hydrophobicity (or polarity). In the serial LC coupling, the retention time of molecules can be used to estimate the polarity of unknown compounds and thus to support their identification.^10^ Unfortunately, there is currently no such estimation available based on retention time in SFC so far. Thus, the retention time does currently not help to rank or exclude candidate compounds for detected features in data evaluation workflows. We are confident that ongoing research in the field of retention mechanisms might help to overcome these challenges in SFC soon. Additionally, this will increase the applicability of SFC and will lead to less laborious method developments. The ultimate goal could be gaining retention models for each stationary phase, which are based on physico‐chemical parameters of separable compounds and be applied estimating retention behavior.

## 3 SFC AS AN ORTHOGONAL TECHNIQUE TO LC

**FIGURE 2:**
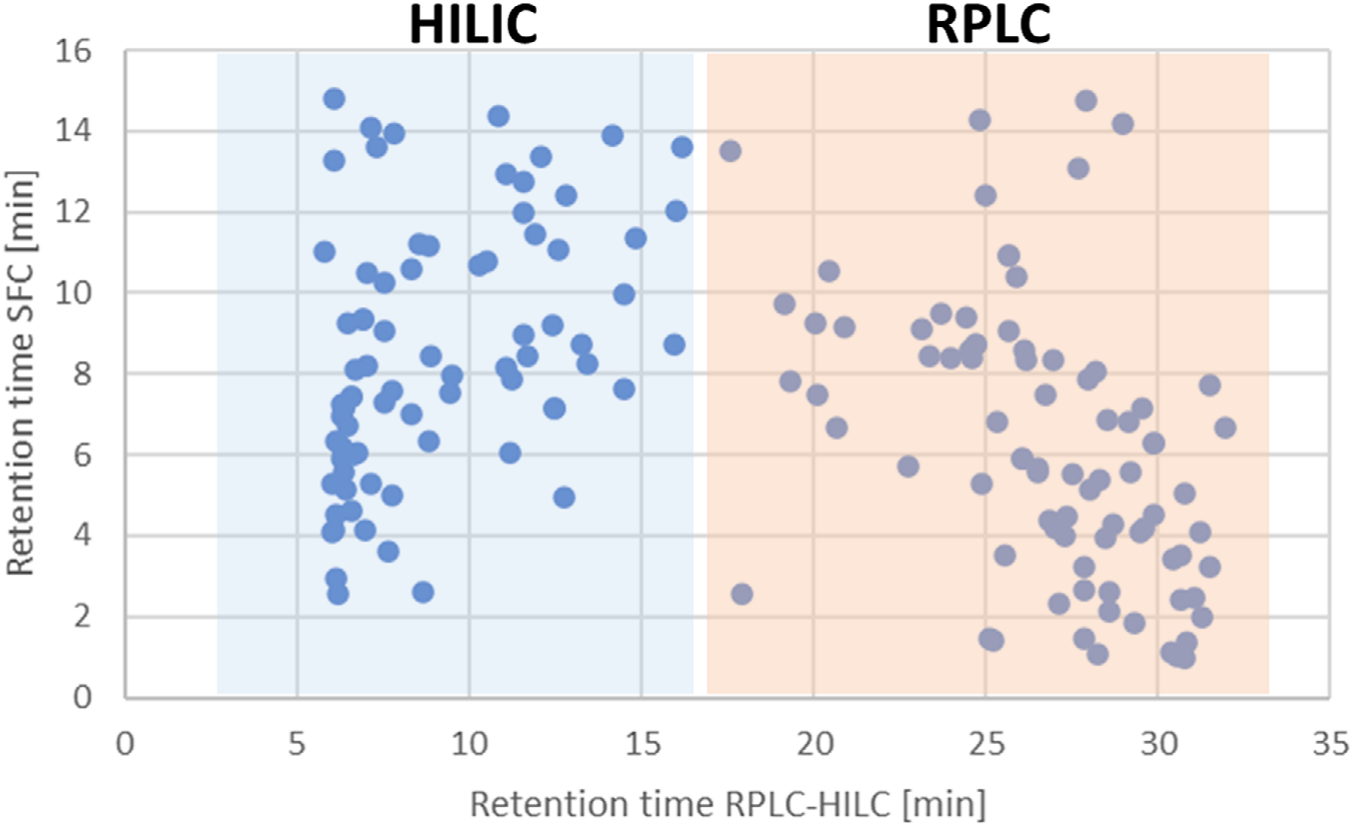
Comparison of retention times of 162 reference compounds, separated by a serial coupling of RPLC and HILIC (*x*‐axis) and SFC (*y*‐axis).

As mentioned above, we compared SFC with a serial coupling of RPLC and HILIC and in 2017, it could be shown that SFC provides the same polarity range of separable compounds as the serial coupling of RPLC and HILIC.^3^ The most remarkable aspect was that this polarity range could be achieved with comparable reproducibility, using only one stationary phase in SFC, instead of two by coupling RPLC and HILIC. Due to obviously different retention mechanisms, the RPLC‐HILIC coupling and SFC allow to achieve comprehensive and orthogonal separations using the same polarity range of analytes.^11^ As shown in Figure 2, SFC is orthogonal and complementary to HILIC and RPLC. This can be used to cross‐evaluate both separation techniques and so to gain additional information about unknown compounds. This is a great advantage for the identification of such unknown compounds in various samples, one of most challenging tasks in NTS.

In the year 2018, as we moved the NTS studies from our academic research group into our new start‐up company for NTS research, NTS education, and NTS consulting,^12^ we established both techniques side by side in the nontarget screening of complex samples. Furthermore, we use them completely equal now.

As already stated, both RPLC‐HILIC and SFC provide highly reproducible and robust separations and are well suitable for routine analysis and "high" sample throughput. Additionally, SFC provides the benefit of efficient separations at increased mobile phase flow rates. Thus, SFC separations may be faster as those with the serial coupling of RPLC and HILIC.

The main reason for that is the characteristic of the mobile phase in SFC, which mainly consists of carbon dioxide with small proportions of organic solvent. Due to the low viscosity and the high diffusion of the mobile phase, SFC separations are very efficient and require less re‐equilibration time compared to HILIC separations, for example. Most instruments (available on market) are capable of achieving an operating pressure up to 400 or 600 bar. This is usually sufficient for moderate flow rates and high modifier content in the mobile phase even when using sub‐2 μm particles in SFC separations. However, for further increasing the efficiency of separations by increasing column length, column diameter, or flow rate, the maximum operational pressure may become a limitation. Of course, columns should also be capable of withstanding the maximum system pressure. A starting point might be here for further optimization or development. In SFC, pressures up to 600 or 1000 bar can be handled nowadays in some systems enabling them in ultra‐high‐performance supercritical fluid chromatography (UHPSFC) without any problems.^13^

The carbon dioxide used for SFC separations is a relatively cheap industrial by‐product. Together with the low consumption of organic solvents, this results in low costs for solvents purchase and disposal. This is an economic but also ecologic benefit in high sample throughput, because the solvents make SFC separations greener and finally more sustainable than LC separations.

The hyphenation of SFC separations with mass spectrometric detection, which is crucial for NTS, can be realized as easy as known from LC and several options to improve sensitivity are available.^14^ The available "high‐flow"‐electrospray ionization sources fit perfect for the combination of SFC and MS detections. To adjust electrospray ionization sources efficient for various SFC‐MS applications, the design of experiments (DoE) strategy can be used in order to optimize ionization parameters in a comprehensive way.^15^ This allows to optimize a large set of parameters in a very systematic way and to find a global optimum for all parameters, using experimental plans and statistical data evaluation. So, it systematically leads to optimal ionization conditions for a broad range of compounds and improved sensitivity in MS detection. Both are main requirements for comprehensive analysis in NTS. Last but not least, SFC is less affected from negative impacts by matrix compounds which impact retention, such as salts.

SFC provides significant benefits over currently used separation techniques, such as the polarity range of separable compounds, speed, and costs per analysis. SFC is an orthogonal technique to RPLC and HILIC and can separate the same compounds. Just as LC, it is also orthogonal to GC, so the combination of these three techniques allows to analyze samples in NTS in a very comprehensive way.

In conclusion, SFC can do many things, which also can be done by LC, but there are several examples besides those shown above, where SFC can be applied to solve problems occurring in LC. These are exactly the fields where SFC should be promoted and be used. With these benefits and its highly reproducible separations, there are so many good reasons for SFC, being widely established as a comprehensive separation technique in routine analysis. In our lab, SFC is already one gold standard separation technique for complex samples. No doubt, SFC has great potential and especially in the new era of mass spectrometric nontarget screening. We are confident that the strengths of SFC will be recognized in several disciplines and we would like to help this technique to become more popular and widely used (not only) in NTS.
